# Associations Among Living Alone, Eating Alone, and Depressive Symptoms: Evidence from a Nationwide Study of South Korean Adults

**DOI:** 10.3390/nu18142379

**Published:** 2026-07-21

**Authors:** Seong-Uk Baek, Jin-Ha Yoon

**Affiliations:** 1 Graduate School, Yonsei University College of Medicine, Seoul 03722, Republic of Korea; 2 Institute for Innovation in Digital Healthcare, Yonsei University Health System, Seoul 03722, Republic of Korea; 3 The Institute for Occupational Health, Yonsei University College of Medicine, Seoul 03722, Republic of Korea; 4 Department of Preventive Medicine, Yonsei University College of Medicine, Seoul 03722, Republic of Korea

**Keywords:** depression, dietary behavior, mental health, single-person households, solitary eating

## Abstract

Background: With the continuous increase in the prevalence of single-person households, growing public health concerns have emerged regarding mental health. This study examined the associations among living alone, eating alone, and depressive symptoms. Methods: We analyzed a nationally representative sample of 23,066 adults from South Korea. Participants were asked whether they lived alone or with other household members and whether they usually ate breakfast, lunch, or dinner alone. Depressive symptoms were assessed using the Patient Health Questionnaire-9. An analysis was conducted to estimate the direct and indirect associations among living alone, eating breakfast, lunch, or dinner alone, and depressive symptoms, using odds ratios (ORs) and 95% confidence intervals (CIs). Results: Among the participants, 12.7% lived alone and 87.3% lived with others. The prevalence of depressive symptoms was 10.6% among those living alone and 4.7% among those living with others. In the analysis, the OR (95% CI) for the total association between solitary eating and depressive symptoms was 1.94 (1.62, 2.35). The OR (95% CI) for the overall indirect association through eating alone was 1.36 (1.24–1.51). Conclusions: This study found an indirect association between living alone and depressive symptoms through solitary eating. Future longitudinal studies are required to establish the temporal ordering among living alone, eating alone, and depressive symptoms and to validate our findings.

## 1. Introduction

Eating together is a social practice that promotes food sharing and strengthens interpersonal bonds. However, eating alone has become increasingly prevalent in recent years [1], which is primarily caused by nuclear family structures, the popularity of fast food, and individualization. The COVID-19 pandemic and related social isolation policies have accelerated this trend [2]. From a public health perspective, this trend may be concerning, as evidence indicates that eating alone is linked to adverse health outcomes, including poor dietary quality [3], metabolic disorders [4], cardiovascular diseases [5], and mental health issues [6,7]. Eating alone was also associated with a greater risk for suicidal death compared to eating with others [8]. In contrast, eating together was linked to greater happiness and reduced risk of depression, anxiety, and stress [9,10].

The prevalence of single-person households or those living alone has increased in South Korea, particularly among young, unmarried, or older adults [11]. Living alone has been linked to various health problems, including increased risks for cardiovascular diseases and all-cause mortality [12,13]. Previous studies have indicated that living alone is associated with mental health problems, including anxiety, depression, and insomnia [14,15,16]. A meta-analysis of longitudinal studies demonstrated that those living alone exhibited a 1.42-times higher odds of depression than those living with other people [17]. Furthermore, living alone, particularly when accompanied by depression, is associated with an elevated risk of suicidal death [18]. Therefore, developing effective strategies to promote good mental health in individuals living alone is an important public health concern.

**Figure 1:**
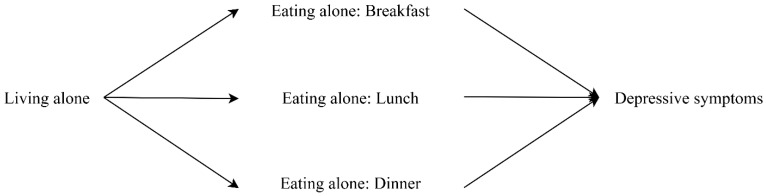
Diagram of the conceptual model in the associations between living alone, eating alone, and depressive symptoms.

Although the exact mechanisms underlying the connection between living alone and depression remain largely unclear, living alone can be associated with social isolation, material deprivation, and unemployment [19], which may contribute to poor mental health. Previous studies exploring underlying factors in this relationship have suggested that factors such as loneliness, social activity engagement, and sleep quality may play crucial roles [20,21,22]. Additionally, an unhealthy diet partially explained the association between living alone and depressive symptoms [23]. Those living alone are more likely to eat alone, which is a major risk factor for depressive symptoms [17]. However, the indirect association between living alone and depressive symptoms through eating alone has not been formally tested. A conceptual model of the associations between living alone, eating alone (breakfast, lunch, and dinner), and depressive symptoms is shown in Figure 1. Based on a nationally representative sample of adults in South Korea, this study aimed to explore the associations among living alone, eating alone, and depressive symptoms.

## 2. Methods

### 2.1. Survey Participants

The study sample was obtained from the Korea National Health and Nutrition Examination Survey (KNHANES), an annual cross-sectional survey that includes a nationally representative sample of people living in South Korea [24]. The study participants were selected based on multistage clustered probability sampling to ensure representation across geographical regions in South Korea [25]. For selected individuals, data were collected using the household-visiting method. Information on depressive symptoms was collected biennially from 2014; thereby, the 2014, 2016, 2018, 2020, and 2022 survey cycles of the KNHANES were included in the present analysis.

**Figure 2:**
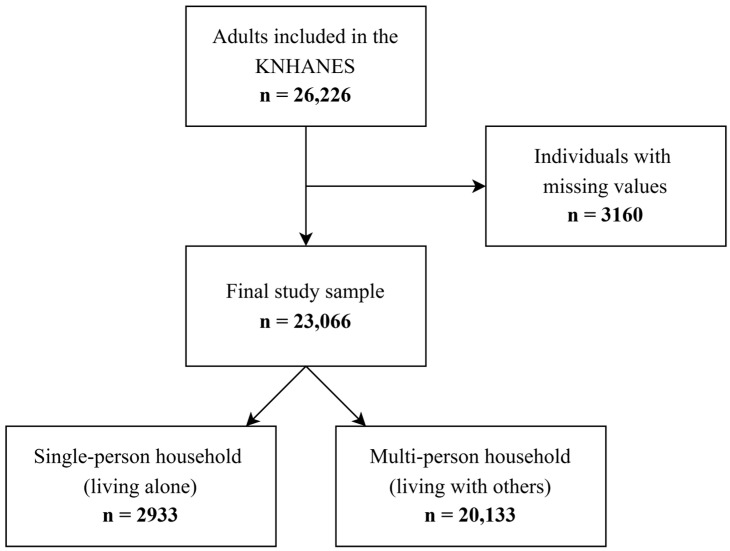
Flowchart of the selection process of the analysis sample.

Figure 2 shows a flowchart of the sample selection process. In total, 26,226 adults aged ≥ 19 years were included in the KNHANES (2014, 2016, 2018, 2020, and 2022). After excluding individuals with missing values (n = 3160), the final study sample comprised 23,066 adults, of whom 2933 lived alone in single-person households and 20,133 resided in multi-person households. The data are available at https://knhanes.kdca.go.kr/ (accessed on 31 December 2024). The study protocols were approved by the Institutional Review Board of the Korea Disease Control and Prevention Agency (2013-12EXP-03-5C; 2018-01-03-P-A; 2018-01-03-2C-A; 2018-01-03-4C-A). Written informed consent was obtained from all study participants. This study was conducted in accordance with the Helsinki Declaration of 1975, as revised in 2024.

### 2.2. Variables

Cohabitation status was assessed using the following question: “How many people are currently living in your household?” Those who responded with one person were classified as living alone, whereas those who responded with two or more people were classified as living with others.

Eating alone was defined as responding “no” to the question of whether breakfast/lunch/dinner was shared with others during the past year. For breakfast, lunch, and dinner, eating alone was dichotomized.

Depressive symptoms were assessed using the Patient Health Questionnaire-9 [26]. The Patient Health Questionnaire-9 has exhibited good validity and reliability in assessing depressive symptoms in Koreans [27]. Among our study sample, the Cronbach’s alpha ranged from 0.78 to 0.81 across the survey years. The composite score ranged from 0 to 27, and individuals with ≥10 of the Patient Health Questionnaire-9 score were categorized as exhibiting depressive symptoms, following the cut-off value used in the previous literature [26].

Covariates included sex, age, income level, education level, employment status, and body mass index. Income levels were divided into four groups (lowest, low, high, and highest) based on the quartile values of annual personal income. Education level was categorized as middle school or below, high school, or college or above based on the highest educational attainment.

### 2.3. Statistical Analysis

#### 2.3.1. Preliminary Analysis

For the preliminary analysis, we first explored the associations between living alone and eating alone and between eating alone and depressive symptoms. Specifically, we examined the associations between living alone and eating alone at each meal (breakfast, lunch, and dinner), and between eating alone at each meal and depressive symptoms. Logistic regression models were used to determine the associations.

#### 2.3.2. Main Analysis

The main analysis investigated the role of eating each meal alone (breakfast, lunch, and dinner) on the association between living alone and depressive symptoms, using the R package “mma” [28]. This approach allows for the estimation of individual indirect association from multiple variables. The associations were presented using odds ratios (ORs). 1000 bootstrap resamplings were performed to compute the 95% confidence intervals (CIs). For sensitivity analysis, we stratified the sample by employment status, considering that employment status can influence whether individuals eat alone or with others. Additionally, factors associated with mental health conditions and dietary quality can differ by employment status [29,30]. Standardized survey weights were applied to enhance the sample representativeness. Statistical analyses were conducted using R (version 4.6.0).

## 3. Results

Table 1 lists the sample characteristics. Among the participants, 12.7% lived alone, and 87.3% lived with others. Compared with those living with others, those living alone were more likely to be female, older, have lower education and income levels, and be unemployed. The prevalence of individuals eating breakfast, lunch, and dinner alone was 66.7%, 54.4%, and 75.2%, respectively. The corresponding figures for individuals living with others were 25.9%, 30.2%, and 15.3%, respectively. The prevalence of depressive symptoms was 10.6% among those living alone and 4.7% among those living with others.

Table 2 presents the results of preliminary analyses. Living alone was positively associated with eating alone for breakfast (OR = 3.40; 95% CI = 3.04, 3.81), lunch (OR = 2.12; 95% CI = 1.90, 2.37), and dinner (OR = 11.74; 95% CI = 10.39, 13.26). The ORs (95% CIs) for the associations of eating alone for breakfast, lunch, and dinner with depressive symptoms were 0.88 (0.76, 1.03), 1.36 (1.17, 1.58), and 1.92 (1.63, 2.28), respectively.

**Figure 3:**
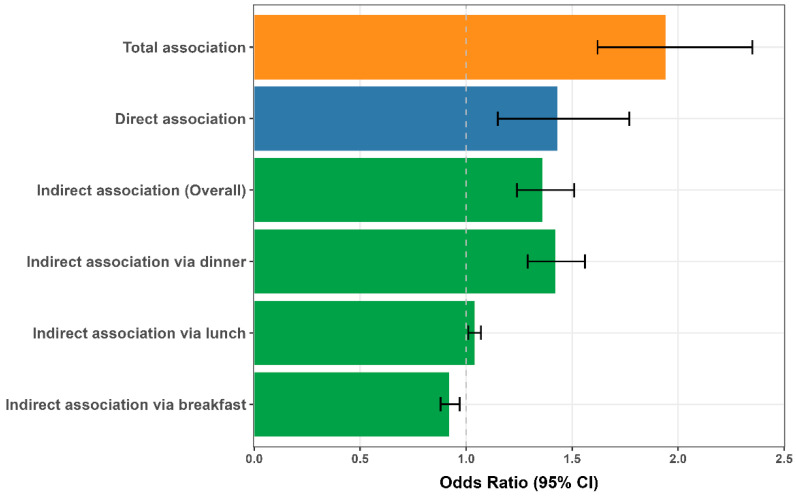
The role of eating alone in the relationship between living alone and depressive symptoms. The model was adjusted for sex, age, education level, income level, employment status, and body mass index.

Figure 3 shows the results of the main analysis. The OR (95% CI) for the total association between eating alone and depressive symptoms was 1.94 (1.62, 2.35). The OR (95% CI) for the direct association between eating alone and depressive symptoms was 1.43 (1.15, 1.77). For individual indirect association, the ORs (95% CIs) for eating alone were 0.92 (0.88, 0.97) for breakfast, 1.04 (1.01, 1.07) for lunch, and 1.42 (1.29, 1.56) for dinner. The overall indirect associations were 1.36 (1.24, 1.51), accounting for 46.2% of the total association between living alone and depressive symptoms (Table S1).

Table S2 shows the results of the stratified analyses. The result shows that the potential role of eating alone was observed among both employed and unemployed individuals.

## 4. Discussion

This study explored the associations among living alone, eating alone, and depressive symptoms among Korean adults. The results showed a positive indirect association between living alone and depressive symptoms through eating alone.

The demographic characteristics of study participants showed substantial differences between those living alone and those living with others. Compared to those living with others, those living alone were more likely to be women, be older, and have lower socioeconomic status, characterized by low education and income levels and unemployment. Such factors may collectively contribute to the high prevalence of mental health problems, such as depressive symptoms among those living alone. Additionally, a previous study reported that widowhood and living alone were associated with a greater risk for loneliness [31], which may be associated with depressive symptoms among those living alone. Therefore, our findings highlight the importance of developing policies and interventions to promote mental health among individuals living alone and facilitate the early identification and intervention of mental health deterioration.

The results of this study are consistent with those of previous studies showing that living or eating alone is linked to depressive symptoms. For instance, living alone is positively associated with the risk of depressive symptoms or suicidal thoughts compared to living with others [17,32,33]. Similarly, eating alone has been linked with depressive symptoms and suicidal thoughts [34,35,36]. Consistent with our findings, a recent study reported that eating alone was indirectly associated with the relationship between living alone and depressive symptoms [6]. However, that study restricted its analysis to participants assessed before the COVID-19 pandemic, given the pandemic-related social distancing measures and the resulting changes in social relationships and working environments [37]. More importantly, it did not examine whether the indirect associations between living alone and depressive symptoms through eating alone differed by meal type (i.e., breakfast, lunch, and dinner). Building on the existing evidence, our study demonstrated that the association between living alone, eating alone, and depressive symptoms may vary by meal type, with eating dinner alone being particularly closely associated with both living alone and depressive symptoms.

Complex mechanisms underlie the association between eating alone and depressive symptoms. Eating together is considered a human behavior that enables food sharing, encourages social interaction, and strengthens communal bonds [38]. In contrast, eating alone is linked to poor dietary quality and irregular meals [3,39]. Additionally, eating alone is associated with poor psychological well-being such as loneliness and stress [40,41]. Such conditions may serve as important pathways linking eating alone and depressive symptoms.

One novel finding of our study is that the indirect association between living alone and depressive symptoms through eating alone differed substantially across meal types. Although the inverse the association between living alone and depressive symptoms through solitary breakfast consumption was unexpected, it may be explained by the inverse association between eating breakfast alone and depressive symptoms. For instance, unlike dinner, which mainly functions as a social activity, breakfast is often considered private time to prepare for the day ahead. Therefore, solitary breakfast eating may be associated with greater autonomy and the alignment with circadian and eating rhythms [42]. These factors may be linked to fewer depressive symptoms [43]. For example, a previous qualitative study suggested that eating alone is related to short meal preparation time and consuming preferred foods. Therefore, eating alone can be less stressful [44]. This aspect is relevant during the morning hours, when people are exposed to time pressure. Supporting this hypothesis, empirical evidence suggests that greater autonomy and voluntary solitude are associated with greater satisfaction with solo dining [45]. Additionally, previous epidemiological studies showed that meal timing that is misaligned with an individual’s circadian rhythm, often referred to as “eating jet lag,” has been associated with poorer mental health outcomes, including depression and anxiety [46,47]. On the other hand, living alone was closely associated with eating dinner alone, which served as the most important pathway of depressive symptoms. Solitary dinner eating is linked to social isolation and poor dietary quality, which may contribute to depression [48]. These findings suggest that meals have distinct characteristics and roles in relation to mental health across breakfast, lunch, and dinner. However, our study did not formally test these underlying mechanisms; the proposed explanations remain speculative and hypothetical. Therefore, the observed associations should be interpreted with caution.

We also identified a direct association between living alone and depressive symptoms. For instance, living alone is linked to social isolation or reduced emotional support, which can contribute to mental health problems [49]. Additionally, previous studies showed that living alone is associated with adopting undesirable lifestyles, including a lack of physical activity, cigarette smoking, and poor sleep quality [50,51,52]. These health-related behaviors may also play an important role.

Certain limitations of this study should be considered when interpreting its findings. First, we could not assert the causal effects of living or eating alone on depressive symptoms owing to the cross-sectional design. Because all variables were measured concurrently, the temporal order among the variables could not be established, and baseline values of eating alone and depressive symptoms could not be considered [53]. For instance, the lack of a clear temporal sequence precludes ruling out reverse causation, whereby depressive symptoms may contribute to social withdrawal and eating alone. Accordingly, we avoided mediation terminology throughout the manuscript, and our findings should be interpreted as evidence of indirect associations identified through path analysis rather than the causal mediation analysis. Future longitudinal studies are warranted to establish the temporal sequence among living alone, eating alone, and depressive symptoms and to better evaluate their relationships. Second, the variables were measured via self-reporting, which raises concerns regarding recall bias. Additionally, while the Patient Health Questionnaire-9 has been widely employed as a tool for measuring depressive symptoms, future studies may benefit from utilizing clinical diagnoses rather than relying on screening tools. Third, due to the lack of information, this study could not encompass important unmeasured confounders, such as the frequency of occupational stress, social activity, psychiatric history, and dietary quality, all of which can influence mental health [54]. Therefore, the omission of these factors may have resulted in residual confounding, potentially leading to an overestimation of the observed associations. Drawing causal inferences requires the absence of unmeasured confounding among main variables. Violation of this assumption may result in biased estimates and preclude causal interpretation [55]. Fourth, eating alone, referred to as honbap in Korea, has become increasingly prevalent in Korean society, particularly among younger adults; it reflects social and cultural shifts toward individualism [56]. Given this cultural context, perceptions and experiences of eating alone may vary substantially across countries, cultures, and generations. Therefore, the generalizability of our findings to other cultural settings may be limited. Fifth, the assessment of eating alone was limited by its dichotomous classification during data collection. Consequently, the frequency and context of eating alone could not be fully captured, and the measure did not distinguish between occasional and frequent eating alone. This limitation may have influenced the observed associations between eating alone, living alone, and depressive symptoms.

Nevertheless, this study enhances the generalizability of its findings by utilizing a nationally representative sample. In addition, our research not only presented novel findings regarding the association between eating alone, living alone, and depressive symptoms but also contributed to the literature by demonstrating how this relationship differs according to meal type across breakfast, lunch, and dinner.

## 5. Conclusions

This study found positive associations among living alone, eating alone, and depressive symptoms among adults in South Korea. While future longitudinal studies are needed to clarify the causal relationship, our study suggests the need for policy efforts to promote the eating behaviors and mental health of individuals living alone. For instance, community meal programs and workplace shared-meal initiatives that provide opportunities for communal dining may help reduce eating alone, facilitate social connectedness and a sense of belonging, and alleviate loneliness among individuals living alone.

## Figures and Tables

**Table 1:** Sample characteristics.

	Overall	Living Alone	Living with Others
	N = 23,066	N = 2933 (12.7%)	N = 20,133 (87.3%)
Sex			
Male	9680 (42.0%)	1132 (38.6%)	8548 (42.5%)
Female	13,386 (58.0%)	1801 (61.4%)	11,585 (57.5%)
Age			
Mean (SD)	51.7 (16.8)	58.5 (18.1)	50.7 (16.4)
Education level			
Middle school or below	6926 (30.0%)	1508 (51.4%)	5418 (26.9%)
High school	7590 (32.9%)	702 (23.9%)	6888 (34.2%)
College or above	8550 (37.1%)	723 (24.7%)	7827 (38.9%)
Income level			
Lowest	5640 (24.5%)	1244 (42.4%)	4396 (21.8%)
Low	5782 (25.1%)	800 (27.3%)	4982 (24.7%)
High	5792 (25.1%)	550 (18.8%)	5242 (26.0%)
Highest	5852 (25.4%)	339 (11.6%)	5513 (27.4%)
Employment status			
Employed	13,749 (59.6%)	1549 (52.8%)	12,200 (60.6%)
Unemployed	9317 (40.4%)	1384 (47.2%)	7933 (39.4%)
Body mass index			
Mean (SD)	24.0 (3.6)	24.2 (3.6)	23.9 (3.6)
Eating alone: Breakfast			
No	15,900 (68.9%)	976 (33.3%)	14,924 (74.1%)
Yes	7166 (31.1%)	1957 (66.7%)	5209 (25.9%)
Eating alone: Lunch			
No	15,397 (66.8%)	1337 (45.6%)	14,060 (69.8%)
Yes	7669 (33.2%)	1596 (54.4%)	6073 (30.2%)
Eating alone: Dinner			
No	17,779 (77.1%)	726 (24.8%)	17,053 (84.7%)
Yes	5287 (22.9%)	2207 (75.2%)	3080 (15.3%)
Depressive symptoms			
No	21,807 (94.5%)	2622 (89.4%)	19,185 (95.3%)
Yes	1259 (5.5%)	311 (10.6%)	948 (4.7%)

SD, standard deviation.

**Table 2:** Logistic regression models on the associations among living alone, eating alone, and depressive symptoms.

Independent Variable	Dependent Variable	OR	95% CI
Pathway: Living alone → Eating alone			
Living alone	Eating alone: Breakfast	3.40	3.04, 3.81
Living alone	Eating alone: Lunch	2.12	1.90, 2.37
Living alone	Eating alone: Dinner	11.74	10.39, 13.26
Pathway: Eating alone → Depressive symptoms			
Eating alone: Breakfast	Depressive symptoms	0.88	0.76, 1.03
Eating alone: Lunch	Depressive symptoms	1.36	1.17, 1.58
Eating alone: Dinner	Depressive symptoms	1.92	1.63, 2.28

OR, odds ratio; CI, confidence interval. The models were adjusted for sex, age, education level, income level, employment status, and body mass index.

## Data Availability

Data is available at https://knhanes.kdca.go.kr/ (accessed on 31 December 2024).

## Supplementary Materials

The following supporting information can be downloaded at: https://www.mdpi.com/article/10.3390/nu18142379/s1, Table S1: Mediating role of eating alone in the relationship between living alone and depressive symptoms; Table S2: Mediating role of eating alone in the relationship between living alone and depressive symptoms stratified by employment status.
