# Supplementary material for: Associations Among Living Alone, Eating Alone, and Depressive Symptoms: Evidence from a Nationwide Study of South Korean Adults
Source: Nutrients. 2026 Jul 21;18(14):2379. doi: 10.3390/nu18142379 (PMC13414851; doi:10.3390/nu18142379)
Supplement: Supplementary file 1 [file nutrients-18-02379-s001.zip › nutrients-4418542-supplementary.pdf]

Table S1 Mediating role of eating alone in the relationship between living alone and depressive symptoms.

	Estimate (95% CI)	OR (95% CI)	Proportion mediated
**Total effect**	0.66 (0.48, 0.86)	1.94 (1.62, 2.35)	
**Direct effect**	0.36 (0.14, 0.57)	1.43 (1.15, 1.77)	
**Indirect effect**			
**Eating alone: Breakfast**	-0.08 (-0.13, -0.03)	0.92 (0.88, 0.97)	-12.2 %
**Eating alone: Lunch**	0.04 (0.01, 0.07)	1.04 (1.01, 1.07)	5.6 %
**Eating alone: Dinner**	0.35 (0.26, 0.44)	1.42 (1.29, 1.56)	52.9 %
**Total**	0.31 (0.21, 0.41)	1.36 (1.24, 1.51)	46.2 %

OR, odds ratio; CI, confidence interval

The model was adjusted for sex, age, education level, income level, employment status, and body mass index.

Table S2 Mediating role of eating alone in the relationship between living alone and depressive symptoms stratified by employment status

	Employed	Unemployed
	**OR (95% CI)**	**OR (95% CI)**
**Total effect**	1.84 (1.43, 2.30)	1.98 (1.53, 2.47)
**Direct effect**	1.47 (1.10, 1.89)	1.35 (1.02, 1.75)
**Indirect effect**		
**Eating alone: Breakfast**	0.93 (0.87, 0.99)	0.91 (0.83, 1.00)
**Eating alone: Lunch**	1.03 (0.99, 1.07)	1.08 (1.03, 1.13)
**Eating alone: Dinner**	1.30 (1.16, 1.47)	1.49 (1.28, 1.73)
**Total**	1.25 (1.11, 1.41)	1.46 (1.25, 1.72)

OR, odds ratio; CI, confidence interval;

The model was adjusted for sex, age, education level, income level, and body mass index.
